# Regulating the Homogeneity of Thiol-Maleimide Michael-Type Addition-Based Hydrogels Using Amino Biomolecules

**DOI:** 10.3390/gels7040206

**Published:** 2021-11-11

**Authors:** Yu Guo, Jie Gu, Yuxin Jiang, Yanyan Zhou, Zhenshu Zhu, Tingting Ma, Yuanqi Cheng, Zongzhou Ji, Yonghua Jiao, Bin Xue, Yi Cao

**Affiliations:** 1 College of Life and Health Sciences, Northeastern University, Shenyang 110819, China; guoyuedu2021@163.com (Y.G.); jizongzhou@163.com (Z.J.); 2 Department of Physics, Key Laboratory of Intelligent Optical Sensing and Manipulation, National Laboratory of Solid State Microstructure, Collaborative Innovation Center of Advanced Microstructures, Ministry of Education, Nanjing University, Nanjing 210093, China; orcs2013@163.com (J.G.); jiangyuxin3053@163.com (Y.J.); zhouyanyan@nju.edu.cn (Y.Z.); zhuzhenshu@163.com (Z.Z.); ttma2748@163.com (T.M.); chengyqnju@163.com (Y.C.)

**Keywords:** hydrogel, reaction kinetics, mechanical homogeneity, peptide

## Abstract

Poly(ethylene glycol) (PEG)-based synthetic hydrogels based on Michael-type addition reaction have been widely used for cell culture and tissue engineering. However, recent studies showed that these types of hydrogels were not homogenous as expected since micro domains generated due to the fast reaction kinetics. Here, we demonstrated a new kind of method to prepare homogenous poly(ethylene glycol) hydrogels based on Michael-type addition using the side chain amine-contained short peptides. By introducing such a kind of short peptides, the homogeneity of crosslinking and mechanical property of the hydrogels has been also significantly enhanced. The compressive mechanical and recovery properties of the homogeneous hydrogels prepared in the presence of side chain amine-contained short peptides were more reliable than those of inhomogeneous hydrogels while the excellent biocompatibility remained unchanged. Furthermore, the reaction rate and gelation kinetics of maleimide- and thiol-terminated PEG were proved to be significantly slowed down in the presence of the side chain amine-contained short peptides, thus leading to the improved homogeneity of the hydrogels. We anticipate that this new method can be widely applied to hydrogel preparation and modification based on Michael-type addition gelation.

## 1. Introduction

Polymer networks of hydrogels are widely used to mimic the extracellular matrix for cell culture in vitro and tissue engineering due to the high water content, the elasticity similar to biological tissue, and the freedom of functional biochemistry [1,2,3,4]. Among various hydrogels, poly(ethylene glycol) (PEG)-based synthetic hydrogels are regarded as one of the most promising candidates because of its hydrophilic, low nonspecific protein adsorption, negative mammalian enzymatic degradation, and controllable polymerization. Moreover, PEG could be easily functionalized since the ends of the polymer chain were capped with hydroxyl, indicating that various gelation reaction can be used for hydrogel polymerization based on PEG such as double-bond polymerization [5,6,7], Michael-type addition [8,9], condensation reaction [10], click chemistry [11,12], and so on [13]. In these gelation reactions based on functional PEG, the Michael-type addition reaction has attracted many interests in preparing hydrogels because it can achieve the highest macromer coupling efficiency based on the reported Michael-type pairs and form a so-called homogeneous polymer network under various conditions without any byproducts [2,8,14,15,16]. However, recent studies showed that these hydrogels based on Michael-type addition reaction were not homogeneous as expected due to the generation of the micro domains caused by the inconsistency of reaction and mixing speed [17,18]. The heterogeneity of hydrogels would lead to inconsistent cellular responses [19] since topographical and mechanical cues may both affect cell behaviors [20,21]. The non-uniform ligand densities and cross-linking gradients in inhomogeneous hydrogels may provide misleading mechanical signals [22,23] when used as cell culture platforms. Since the stem cells behaviors and fate could be obviously affected by the micro-zone mechanical environments, the misleading signals from the inhomogeneous hydrogels may lead to the unexpected cell responses [24].

In order to prepare homogeneous hydrogels, many efforts have been made to decrease the reaction rates of maleimide- and thiol-modified polymers and slow down the gelation kinetics of Michael-type addition-based hydrogels [25,26]. Several effective approaches including decreasing the reacting temperature, lowering the polymer weight percentage and pH have been developed to reduce the reaction kinetics [17,27]. Recently, negative charges peptides have been introduced as crosslinkers to increase the pKa of thiol groups without any effects on cell viabilities [8,28]. Competitive binding molecules or scrambled molecules that can form complex with the reactants were also introduced to decrease the availability of reactant during the gelation [24]. However, these methods still suffer deficiencies owning to that these methods are often cytotoxic for cell encapsulation or gelation in vivo. The scrambled molecules may even weak the strength of hydrogels by reducing the cross-linking density due to the irreversible interactions with reactant. The reliable and cost-effective method to slow gelation kinetics and prepare homogeneous hydrogels based on Michael-type addition still remains challenging.

Here, we reported a new kind of method to prepare homogenous poly(ethylene eglycol) hydrogels based on Michael-type addition. By introducing the short peptides containing amino group at the side chain into the gelation system, the homogeneity of the crosslinking distribution and mechanical property for the hydrogels were significantly enhanced at the nanoscale. Moreover, the resulted homogeneous hydrogels exhibited reliable mechanical properties at the macroscale with the excellent biocompatibility remained. Further studies indicated that the improved homogeneity was mainly attributed to the slowing down of the reaction rate of thiol- and maleimide- terminated PEG and gelation rates of thiol- and maleimide-terminated PEG-based hydrogels. We expect that this approach of generating homogenous hydrogels with side chain amine-contained short peptides can be widely used in the preparation and modification of hydrogels based on Michael-type addition.

## 2. Results and Discussion

### 2.1. Design and Generation of the Homogeneous Poly(Ethylen Eglycol) Hydrogels

**Figure 1:**
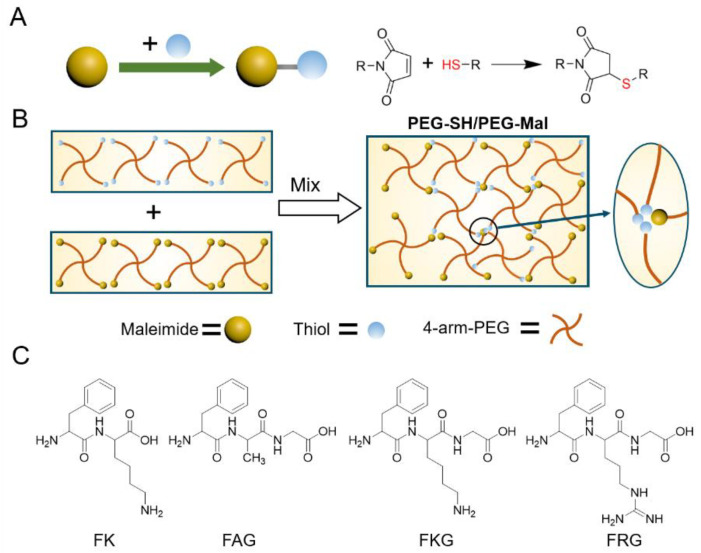
Schematic for the reaction of thiol and maleimide, the gelation of PEG-SH and PEG-Mal, and chemical structures of the side chain amine-contained short peptides. (**A**) Schematic for the Michael addition of the thiol to the maleimide. (**B**) Schematic of the generation of inhomogeneous hydrogels based on the PEG-SH and PEG-Mal. (**C**) Chemical structures of the side chain amine-contained short peptides (FK, FKG and FRG). FAG was selected for comparison.

The hydrogels based on maleimide-terminated four-armed polyethylene glycol (Mw: 20 kDa, named as PEG-Mal) and thiol-terminated four-armed polyethylene glycol (Mw: 20 kDa, named as PEG-SH) were used as the model gel. As illustrated in Figure 1A, thiol and maleimide react through the rapid propagation of the thiolate onto the vinyl ring of the maleimide and the sequential chain-transfer of the hydrogen [29,30,31,32]. The reaction is fast and the product is stable. As a result, the intensive mixing of thiol and maleimide was limited during the gelation process, leading to the generation of micro domains formed by unreacted functional groups (Figure 1B). Interestingly, we found that some peptides containing amine at side chain can reduce the reaction and gelation kinetics of maleimide-terminated and thiol-terminated PEG by being added in precursors, thus increasing the crosslinking and mechanical homogeneity of the hydrogels. As shown in Figure 1C, Phenylalanine-lysine (FK), Phenylalanine-lysine-glycine (FKG), and Phenylalanine-arginine-glycine (FRG) were used to act as the side chain amine-contained peptides while Phenylalanine-alanine-glycine (FAG) was set for comparison (Figure 1C). The hydrogels prepared in the absence and presence of the peptides are denoted as PEG-SH/PEG-Mal and PEG-SH/PEG-Mal/Pep hydrogels hereafter.

### 2.2. Crosslinking Homogeneity of the PEG-SH/PEG-Mal/Pep Hydrogels

**Figure 2:**
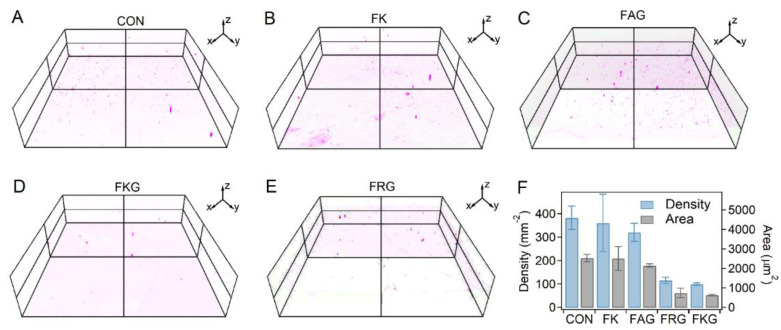
Spatial detection of unreacted thiol in PEG-Mal/PEG-SH and PEG-Mal/PEG-SH/Pep hydrogels. (**A**−**E**) Spatial distribution of unreacted thiol in PEG-Mal/PEG-SH/Pep hydrogels detected using LCFM. The unreacted PEG-SH was labeled with the thiol turn-on fluorescence probe. The hydrogels prepared without peptides was set as control. The red spots correspond to the locations of unreacted thiol, and the size of the scanning space was 1272 × 1272 × 300 μm. (**F**) Density and area of the fluorescent spots from the projected images of the three-dimensional constructs in the *Z*-axis direction for different hydrogels. Values represent the mean and standard deviation (*n* = 4–5).

The distribution of unreacted thiol in PEG-SH/PEG-Mal hydrogels in the presence of side chain amine-contained short peptides were examined in order to investigate the homogeneity of crosslinking in the hydrogel. Free thiol in the hydrogels were labeled with thiol-selective fluorogenic probes [33], whose fluorescence intensity would enhance for more than 100 times after reacting with thiol. Then, the hydrogels were investigated using a laser confocal fluorescence microscopy (LCFM) to detect the spatial distribution of the free thiol. As shown in Figure 2A–E, the fluorescent spots could be considered as the bulk cracks of the hydrogels. Obviously, the fluorescent spots in the hydrogels prepared in the presence of FKG and FRG decreased comparing to those in the control groups. In contrast, more fluorescent spots were observed in the hydrogels prepared with FK and FAG peptides, indicating that the generation of more cracks. Moreover, the fluorescent spots in PEG-SH/PEG-Mal hydrogels prepared without peptides were more disordered than that of PEG-SH/PEG-Mal/FKG and PEG-SH/PEG-Mal/FRG hydrogels. The density and area of the fluorescent spots from the projected images of the three-dimensional constructs in the *Z*-axis direction were shown in Figure 2F. The density and area of the fluorescent spots in hydrogels prepared with FKG and FRG peptides were ~70–75% lower than that of hydrogels prepared without peptides. In contrast, the FAG peptide only leads to ~20% decrease of the fluorescent spots while the FK peptide entirely cannot decrease the fluorescent spots (Figure 2F). The higher density and area of the fluorescent spots in PEG-SH/PEG-Mal/FK and PEG-SH/PEG-Mal/FRG hydrogels can be attributed the higher density of free thiol, indicating the ignorable effects of FK and FRG on improving homogeneity of crosslinking of the hydrogels. Furthermore, the effects of the molar ratios of peptide and PEG-Mal (FKG:PEG-Mal) on the distribution of fluorescent spots were studied (Figure S1). For hydrogels prepared at the FKG:PEG-Mal ratio of 1:4 and 2:4, the fluorescent spot density and area were similar and about 76–80% lower than those of control groups. For hydrogels at the FKG:PEG-Mal ratio of 4:4, the fluorescent spot density and area slightly increased and were about 68% lower than those of control groups.

To further determine the unreacted thiol remaining in the hydrogels, the amounts of free thiol were also quantified with 5,5′-Dithiobis-(2-nitrobenzoic acid) (DTNB) [34] (Figures S2 and S3). As shown in Figure S2A,B, the OD_412nm_ values of the PEG-SH/PEG-Mal/FKG and PEG-SH/PEG-Mal/FRG hydrogels decreased for more than 75% compared to that of PEG-SH/PEG-Mal hydrogels. The OD_412nm_ value of the PEG-SH/PEG-Mal/FAG hydrogel decreased for less than 25%, whereas that of the PEG-SH/PEG-Mal/FK hydrogel was almost the same with that of PEG-SH/PEG-Mal hydrogels, similar with the detecting of the thiol distribution. The amounts of unreacted thiol for hydrogels at different FKG:PEG-Mal ratios also exhibited the similar trend with that of spatial distribution (Figure S3). These results suggested that the presence of FKG and FRG can effectively enhance the homogeneity of crosslinking and reduce the amount of unreacted thiol in hydrogels at the same time, while FAG and FK caused slight/ignorable effects.

### 2.3. Mechanical Homogeneity of the PEG-SH/PEG-Mal/Pep Hydrogels

**Figure 3:**
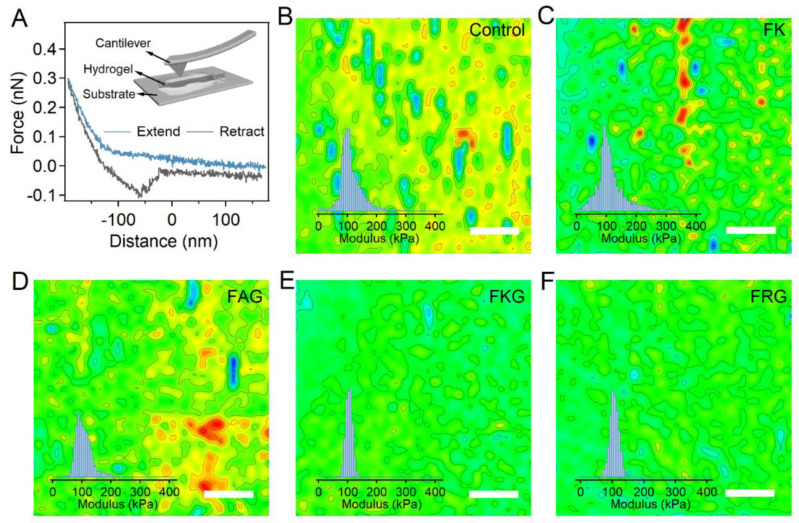
Mechanical homogeneity of PEG-Mal/PEG-SH and PEG-Mal/PEG-SH/Pep hydrogels. (**A**) Typical force–distance curve of the IT-AFM experiments. The inset corresponds to the schematic illustration of the IT-AFM experiments on hydrogel samples. The hydrogels were immersed in PBS on the glass substrates. The cantilever tip approached the hydrogel surface and then retracted, during which process the force–distance curves were recorded. The modulus of the hydrogel surfaces was calibrated from the force–distance curves based on the Hertz model. (**B**−**F**) Two-dimensional Young’s modulus distributions of hydrogel surfaces determined by AFM for PEG-SH/PEG-Mal (**B**), PEG-SH/PEG-Mal/FK (**C**), PEG-SH/PEG-Mal/FAG (**D**), PEG-SH/PEG-Mal/FKG (**E**), and PEG-SH/PEG-Mal/FRG (**F**) hydrogels. The scale bar is 1.0 μm. Insets correspond to the histograms of Young’s modulus.

In order to study the mechanical homogeneity of the PEG-Mal/PEG-SH/Pep hydrogels, the Young’s modulus of hydrogel surface was quantified with nanoindentation based on the atomic force microscopy (IT-AFM) with submicrometer spatial resolution. Typically, hydrogels were carefully transferred to a flat glass coverslip in the PBS solution. The cantilever approached the surface of hydrogels at a constant speed of 2 μm s^−1^ and then retracted at the same speed (Figure 3A). The force and distance during the approaching and retracting process were recorded. Then, the Young’s modulus of the hydrogel surface was calculated by fitting the approaching traces of the force–displacement curves with the Hertz model. As shown by the representative maps (Figure 3B–F, 40 × 40 pixels), the spatial distribution of Young’s modulus for PEG-SH/PEG-Mal/FKG and PEG-SH/PEG-Mal/FRG hydrogels was more pronounced than those of PEG-SH/PEG-Mal hydrogel, suggesting the improvement of mechanical homogeneities. In contrast, the Young’s modulus of PEG-SH/PEG-Mal/FAG and PEG-SH/PEG-Mal/FK hydrogels were disordered, indicating the ignorable improvements on mechanical homogeneities. The histogram distribution and scatter diagram of Young’s modulus based on four to six areas for different hydrogels were summarized in the insets of Figure 3B–F and Figure S4. The Young’s modulus of the PEG-SH/PEG-Mal, PEG-SH/PEG-Mal/FK, PEG-SH/PEG-Mal/FAG, PEG-SH/PEG-Mal/FKG, and PEG-SH/PEG-Mal/FRG hydrogels were 95.1, 93.2, 97.1, 104.3, and 108.9 kPa, respectively. The average Young’s modulus of the PEG-SH/PEG-Mal/FKG and PEG-SH/PEG-Mal/FRG hydrogels slightly increased due to the efficient crosslinking of thiol and maleimide. Furthermore, the standard deviations (SD) of the Young’s modulus for PEG-SH/PEG-Mal/FKG and PEG-SH/PEG-Mal/FRG hydrogels were much smaller than those for PEG-SH/PEG-Mal/FK and PEG-SH/PEG-Mal/FAG hydrogels, consistent with two-dimensional distribution of Young’s modulus (Figure S4F). The Young’s modulus distributions of the hydrogels prepared at different FKG:PEG-Mal ratios (0:1, 1:4, 2:4, and 4:4) were also evaluated (Figures S5 and S6). Obviously, addition of different ratios of FKG can increase the mechanical homogeneity of the hydrogels. Interestingly, no obvious difference of the standard deviation was observed for hydrogels prepared at varied FKG:PEG-Mal ratios, suggesting that the FKG peptide can enhance the mechanical homogeneity of the hydrogels effectively even at low concentrations. All these results suggested that the introduction of the side chain amine-contained short peptides can significantly improve the mechanical homogeneity of hydrogels.

### 2.4. Mechanical and Bulk Properties of the PEG-SH/PEG-Mal/Pep Hydrogels

**Figure 4:**
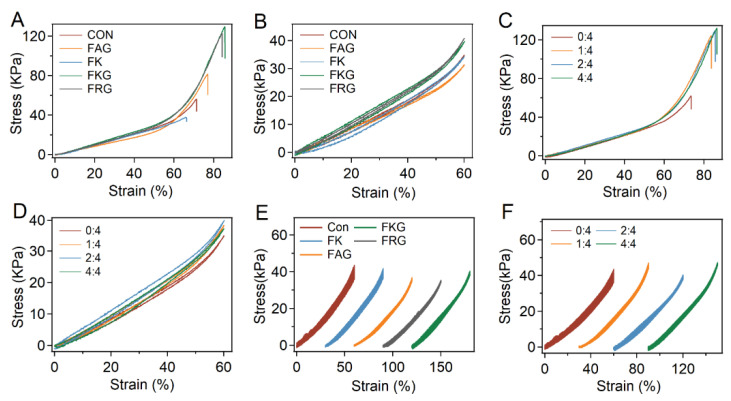
Compressive mechanical properties of PEG-SH/PEG-Mal/Pep hydrogels. (**A**,**B**) Typical stress–strain (**A**) and compression–relaxation (**B**) curves of different PEG-SH/PEG-Mal/Pep hydrogels. The PEG-SH/PEG-Mal hydrogel was set as the control group. (**C**,**D**) Typical compressive stress–strain (**C**) and compression–relaxation (**D**) curves of PEG-SH/PEG-Mal/FKG hydrogels prepared at different FKG:PEG-Mal ratios (0:4, 1:4, 2:4, and 4:4). (**E**,**F**) Stress–strain curves of 100 consecutive compression–relaxation cycles for different PEG-SH/PEG-Mal/Pep hydrogels (**E**) and PEG-SH/PEG-Mal/FKG hydrogels at different FKG:PEG-Mal ratios (**F**).

Next, the compressive mechanical properties of the PEG-SH/PEG-Mal/Pep hydrogels were studied in details. As shown in Figure 4A, the fracture strains of PEG-SH/PEG-Mal/FKG and PEG-SH/PEG-Mal/FRG hydrogels were higher than that of hydrogels prepared without peptides, probably due to the less cracks as indicated by unreacted thiol detection. In contrast, the fracture strains of PEG-SH/PEG-Mal/FAG and PEG-SH/PEG-Mal/FK hydrogels were similar to that of PEG-SH/PEG-Mal hydrogels. The Young’s modulus and toughness of the hydrogels were summarized in Figure S7A. The Young’s modulus slightly increased from ~37.9 kPa of PEG-SH/PEG-Mal gels to ~47.6 and ~48.3 kPa of PEG-SH/PEG-Mal/FKG and PEG-SH/PEG-Mal/FRG hydrogels due to the higher reaction efficiency of thiol and maleimide. Moreover, the toughness significantly improved from ~16.8 kJ m^−3^ of PEG-SH/PEG-Mal hydrogels to more than 28.3 kJ m^−3^ of PEG-SH/PEG-Mal/FKG and PEG-SH/PEG-Mal/FRG hydrogels because of the larger fracture strains. However, the enhancements of Young’s modulus and toughness were moderate for PEG-SH/PEG-Mal/FAG hydrogels and almost ignorable for PEG-SH/PEG-Mal/FK hydrogels, similar with the trend of homogeneity of the hydrogels. The compress–relaxation of the hydrogels were also studied in Figure 4B and no obvious hysteresis was observed in all the hydrogels. Moreover, the mechanical properties of the PEG-SH/PEG-Mal/FKG hydrogels at different FKG:PEG-Mal ratios were also investigated (Figure 4C,D). The PEG-SH/PEG-Mal/FKG hydrogels at various FKG:PEG-Mal ratios exhibited obviously enhanced facture strains and slightly increased Young’s modulus compared to PEG-SH/PEG-Mal hydrogels (Figure 4C and Figure S7B). Meanwhile, the compression–relaxation was not affected by FKG:PEG-Mal ratios (Figure 4D).

Besides the compressive mechanical properties of the hydrogels, the recovery properties were studied by applying continuous compression–relaxation cycles to the hydrogels without any waiting time between each cycle. As shown in Figure 4E, the stress–strain curves of PEG-SH/PEG-Mal/FKG and PEG-SH/PEG-Mal/FRG were almost superimposable, while that of PEG-SH/PEG-Mal hydrogels gradually shifted. The maximum stress of PEG-SH/PEG-Mal hydrogels decreased to ~84% after 100 compression–relaxation cycles (Figure S8A). Yet the maximum stress of PEG-SH/PEG-Mal/FKG and PEG-SH/PEG-Mal/FRG hydrogels remained more than 95%, suggesting that the improved homogeneity of the hydrogels could lead to outstanding performance of fast recovery. The maximum stress of PEG-SH/PEG-Mal/FK and PEG-SH/PEG-Mal/FAG reached 86 and 92% after the 100 compression–relaxation cycles, indicating the ignorable and moderate increasements of recovery properties brought by FK and FAG peptides. The effects of different FKG:PEG-Mal ratios (0:4, 1:4, 2:4, and 4:4) on the recovery properties of hydrogels were shown in Figure 4F and Figure S8B. The maximum stress of PEG-SH/PEG-Mal/FKG at the FKG:PEG-Mal ratios of 1:4, 2:4, and 4:4 after 100 compression–relaxation cycles were in the range of 93–97%. All the hydrogels at different FKG:PEG-Mal ratios exhibited improved recovery performance since that the FKG peptide can increase the hydrogel homogeneity even at low concentrations. All these measurements confirmed the reliable mechanical performance and fast recovery rate of hydrogels prepared in the presence of side chain amine-contained short peptides.

Moreover, the swelling ratio and porosity of PEG-SH/PEG-Mal and different PEG-SH/PEG-Mal/Pep hydrogels were all about 3.6 and 95% (Figure S9). All the hydrogels exhibited similar porous microstructures at micrometer scales as indicated by scanning electron microscope (SEM) images (Figures S10 and S11). The similar swelling ratios, porosity, and microstructures of all the hydrogels indicated that the bulk properties were not affected by the introducing of peptides. Besides, more than 90% peptide can be removed during the dialysis of hydrogels, indicating the absence of peptides in the resulted hydrogels (Figure S12).

### 2.5. Biocompatibility of the PEG-SH/PEG-Mal/Pep Hydrogels

Since thiol-maleimide-based PEG hydrogels are widely used in tissue engineering, the cell culture performance of the PEG-SH/PEG-Mal/Pep hydrogels were also investigated in order to study the biocompatibility. Human amniotic mesenchymal stem cells (HAMSC) and Human hepatocellular carcinoma cells (Huh7) were chosen to act as the model cells to be cultured on the hydrogels. The cell morphology and viability after being cultured for 60 or 24 h were determined using live/dead cell staining (Figures S13 and S14). As shown in Figures S13A and S14A, the morphologies of both HAMSC and Huh7 cells cultured on different PEG-SH/PEG-Mal/Pep hydrogels were the same as those of cells cultured on PEG-SH/PEG-Mal hydrogels (Control) or cell culture plates (Blank). The amounts of dead cells with compromised membranes were almost ignorable and the living cells with high enzymatic activity spread all over the hydrogels, indicating that the cell spreading was also not affected by the regulation of peptides. The cell viabilities of HAMSC and Huh7 cells on all the hydrogels were all higher than 95% according to the live and dead cell counting (Figures S13B and S14B).

### 2.6. Reaction and Gelation Kinetics of the PEG-SH/PEG-Mal/Pep Hydrogels

**Figure 5:**
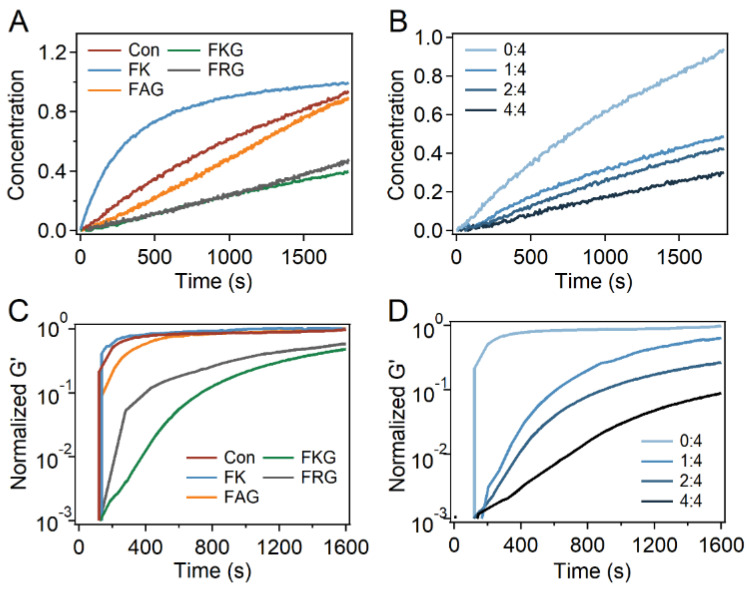
Reaction and gelation kinetics of the PEG-Mal/PEG-SH/Pep hydrogels. (**A**,**B**) Reaction kinetics of different PEG-Mal/PEG-SH/Pep hydrogels (**A**) and PEG-Mal/PEG-SH/FKG hydrogels at different FKG:PEG-Mal ratios (**B**) over time. The ratios of FKG and PEG-Mal were 0:4, 1:4, 2:4, and 4:4. (**C**,**D**) Gelation kinetics of different PEG-Mal/PEG-SH/Pep hydrogels (**C**) and PEG-Mal/PEG-SH/FKG hydrogels at different FKG:PEG-Mal ratios (**D**) monitored by oscillation rheology over time at 25 °C. The ratios of FKG and PEG-Mal were 0:4, 1:4, 2:4, and 4:4.

At last, the reaction and gelation kinetics of PEG-SH and PEG-Mal in the presence of different peptides were investigated. As indicated by the UV spectroscopy, the absorbance at ~300 nm decreased with the reaction of PEG-SH and PEG-Mal proceeding in the presence of different peptides, indicating that the generation of the maleimide-thiol adduct could be monitored using OD_300nm_ (Figure S15A–G). The generation of the maleimide-thiol adduct vs. time in the presence of different peptides was calculated and used to identify the reaction kinetics (Figure 5A and Figure S15H,I). The average reaction rates under the modification of different peptides and FKG:PEG-Mal ratios were summarized in Figure S16A,B. Obviously, the reaction rates of PEG-SH and PEG-Mal was significantly decreased in the presence of FKG and FRG peptides compared to that of the control group. In contrast, slight decrease of the reaction rate in the presence of FAG was observed, indicating that the peptide containing only terminal amine has limited effects on the reaction kinetics of thiol and maleimide. It is worth mentioning that the reaction rate in the presence of FK peptide obviously enhanced instead of decreasing. Besides, the reaction kinetics of PEG-SH and PEG-Mal at different FKG:PEG-Mal ratios (0:1, 1:1, 1:2, and 1:4) was also studied (Figure 5B). The amplitudes of slowing down increased with the scale up of the FKG:PEG-Mal ratio, suggesting that the reaction rate could be further regulated using the FKG:PEG-Mal ratio.

Furthermore, gelation kinetics of the PEG-Mal/PEG-SH/Pep hydrogels in the presence of different peptides and FKG:PEG-Mal ratios were also studied by oscillation rheology over time at room temperature (Figure 5C,D). As shown by the normalized storage modulus (G’) of hydrogels after the mixing of PEG-SH and PEG-Mal, the gelation rates significantly decreased in the presence of FKG and FRG peptides (Figure 5C). The gelation rates in the presence of FAG slightly decreased while the gelation rate in the presence of FK peptide was almost the same as that of control groups. Moreover, the gelation kinetics can also be adjusted with molar ratios of peptide and PEG-Mal (Figure 5D). Similar with that of reaction kinetics, the gelation rate decreased with the increase of FKG:PEG-Mal ratios. Moreover, the summarized average gelation rates suggested the same trends (Figure S16C,D). All these results suggested that the reaction and gelation kinetics of thiol and maleimide were regulated down by the addition of side chain amine-contained peptides.

It is worth noting that even though it has been found that the side chain amine-contained peptides can significantly reduce the reaction and gelation kinetics of the thiol- maleimide-based hydrogels, the detailed mechanism during the process remains unknown, which will be our next evocator. One possible explanation is that the thiol exchange in the maleimide-thiol complex would be significantly enhanced under the catalysis of biomolecule-amine, leading to the increased dynamic property of maleimide-thiol reaction as well as decreased reaction rates and gelation kinetics [35].

## 3. Conclusions

In summary, we demonstrated a new kind of method to prepare homogeneous poly(ethylene glycol) hydrogels based on Michael-type addition reaction using peptides with amino groups at the side chain. By adding the short peptides in the hydrogel precursors, the crosslinking and mechanical homogeneity of the hydrogels were significantly improved. Moreover, the compressive mechanical properties and recovery property of hydrogels prepared under the regulation of the peptides were more reliable than those prepared without any peptides, while the excellent biocompatibility remained unchanged. At last, the reaction and gelation kinetics based on the thiol-maleimide adducts were found to be regulated down, leading to the adequately mixing of reactants and formation of homogenous hydrogel networks. We expect that this new approach to generate homogeneous hydrogels may find broad applications in the preparation and modification of hydrogels based on Michael-type addition.

## 4. Materials and Methods

Materials: Maleimide-terminated 4-armed polyethylene glycol (Mw: 20 kDa) and thiol-terminated 4-armed polyethylene glycol (Mw: 20 kDa) were purchased from Sinopeg, China. FK, FAG, FRG, and FKG peptides was purchased form GL Biochem, China. The thiol-selective fluorescent probe was synthesized in the lab. The HAMSC and Huh7 cell line were purchased from Cell Bank of the Chinese Academy of Sciences (Shanghai, China). The calcein-AM and propidium iodide (PI) double staining kit (cat: KGAF001) was purchased from Keygen, China. Unless specially stated, all the other regents were purchased form Aladdin, China.

Preparation of PEG-SH/PEG-Mal and PEG-SH/PEG-Mal/Pep hydrogels: PEG-Mal and PEG-SH were dissolved in PBS (10 mM, pH = 6.8) to the concentration of 3.5 mM, respectively. For the preparation of PEG-SH/PEG-Mal/Pep hydrogels, the peptide was dissolved in the PEG-Mal solutions to the concentration of 1.75 mM. Then, PEG-SH solution was mixed with the PEG-Mal/peptide solution in equal volume rapidly. The transparent hydrogels formed after the mixing. For the preparation of PEG-SH/PEG-Mal/FKG hydrogels, three different peptide concentrations were used (0.875, 1.75, and 3.50 mM). The resulted hydrogels were dialyzed in ddH_2_O for 24 h to remove the peptide and unreacted PEG. The PEG-SH/PEG-Mal hydrogels were prepared in the absence of peptide with the same method.

LCMF experiments: The PEG-SH/PEG-Mal and PEG-SH/PEG-Mal/Pep hydrogels were prepared as described above. Then, the prepared hydrogel was immersed in the solution of the thiol selective probe (1 mg mL^−1^) synthesized according to the protocol reported by M.G.Finn [33], allowing reacting between the fluorescent probe and free thiol in the hydrogel. The unreacted fluorescent probes in the hydrogels were removed by dialysis in ddH_2_O for 24 h. Lastly, the hydrogels were scanning with a laser confocal fluorescence microscopy (Olympus FV3000, Japan) with the scanning size of 1272 × 1272 × 300 μm. The three-dimensional reconstructions were completed with the commercial software provided by Olympus (FV31S-SW).

Nanoindentation measurement based on the atomic force microscopy (IT-AFM): Typically, the hydrogel film is stuck on the surface of the glass substrate in PBS (10 mM, pH = 7.4). The AFM nano-indentation experiments were performed using a commercial AFM (JPK, Nanowizard IV, Berlin, Germany). The D type of MLCT cantilevers (Bruker, Germany; half-open angle: θ < 20°, tip radius: 20 nm) were used for all experiments. The spring constant of the cantilever (50–60 pN nm^−1^) was calibrated in the solvent for each experiment prior to the measurements. The maximum loading force was set at 500 nN. All AFM experiments were carried out at room temperature. The cantilever was brought to the samples with the constant speed of 2 μm s^−1^ until the loading force reached 300 nN. Then, the cantilever was retracted and moved to another spot for the next cycle. The force–distance curves during the extending and retracting progress were recorded. By fitting the approaching curve to the Hertz model (1), the Young’s modulus of the hydrogels was obtained.

(1) $$F\left(h\right)=\frac{2}{\pi }\tan\alpha \frac{E_{gel}}{1-v_{gel}^{2}}h^{2}\;\;$$

In which F is the stress of the cantilever, h is the depth of the hydrogel pressed by the cantilever tip, *α* is the half angle of the tip, *E* is the Young’s modulus, and *v* is the Poisson ratio. We chose *v* = 0.5 in our calculation. Typically, 5–8 such regions (5 μm × 5 μm, 400 pixels) were randomly selected on each sample to make the elasticity histogram. The two-dimensional modulus distributions were reconstructed using Origin.

Compressive test: Mechanical measurements of the hydrogels were carried out in air using a tensile-compressive tester (instrument 5944 with 2 kN sensor). In the compression test, the rate of deformation was maintained at 5 mm min^−1^. In the compress–relaxation cycle tests, the rate of compression was also kept at 25 mm min^−1^ and each hydrogel was repeatedly compressed for 100 times. The stress (σ) was calculated as the compression force divided by the cross section of the hydrogels, which was monitored by a side view CCD camera during the compression process. The toughness was calculated by the integration of the area below the compression force–distance curves until fracture point. The Young’s moduli were the approximate linear fitting values of the stress–strain curves in the strain range of 0–20%.

Reaction kinetics measurements: The reaction rate of PEG-SH and PEG-Mal was monitored by UV-vis spectra. The UV absorbance vs. time at 300 nm for the mixture of PEG-SH (0.4 mM), PEG-Mal (0.4 mM), and peptide (0.2 mM) in PBS (10 mM, pH = 6.8) was recorded using an ultraviolet spectrophotometer (V550, JASCO Inc., Japan) to monitor the concertation decrease of PEG-Mal. For the reaction in the presence of FKG peptide, three different peptide concentrations were used (0.1, 0.2, and 0.4 mM). Then, the concertation of PEG-Mal and PEG-SH adducts vs. time were calculated according the calibration curves and used to indicate the reaction kinetics of maleimide and thiol. The cuvette width was 1 cm and the bandwidth was set as 0.2 nm.

Gelation kinetics measurements: Typically, the solutions of PEG-Mal/peptide (C_PEG-Mal_ = 3.5 mM) containing different peptide (1.75 mM) and 4-armed PEG-SH (C_PEG-SH_ = 3.5 mM) were mixed and transferred to the rheometer plate of the Thermo Scientific Haake RheoStress 6000 with a pipette quickly. For the gelation in the presence of FKG peptide, three different peptide concentrations were used (0.875, 1.75, and 3.50 mM). Then, the rheology experiments were carried out using a time dependent mode with frequency of 1 Hz and strain of 0.1% immediately (geometry: 1°/20 mm of cone and plate; gap: 0.05 mm; temperature: 20 °C).

## Data Availability

All data are available in the main text or the Supplementary Information.

## Supplementary Materials

The following are available online at https://www.mdpi.com/article/10.3390/gels7040206/s1, Figure S1: Spatial detection of unreacted thiol in PEG-Mal/PEG-SH/FKG hydrogels at different FKG:PEG-Mal ratios. Figure S2: Spatial detection of unreacted thiol in PEG-Mal/PEG-SH/FKG hydrogels at different FKG:PEG-Mal ratios. Figure S3: Detection of free thiol in PEG-Mal/PEG-SH/FKG hydrogels at different FKG:PEG-Mal ratios using DTNB. Figure S4: Typical scatter diagrams and standard deviation summary of Young’s modulus from IT-AFM measurements for different PEG-Mal/PEG-SH/Pep hydrogels. Figure S5: Mechanical homogeneity of the PEG-Mal/PEG-SH/FKG hydrogels at different FKG:PEG-Mal ratios. Figure S6: Typical scatter diagrams and standard deviation summary of Young’s modulus from IT-AFM measurements for PEG-Mal/PEG-SH/FKG hydrogels at different FKG:PEG-Mal ratios. Figure S7: Summarized Young’s modulus and toughness of the PEG-SH/PEG-Mal/Pep hydrogels. Figure S8: Normalized maximum stress for different hydrogels in 100 compression-relaxation cycles. Figure S9: Swelling ratios and porosities of different hydrogels. Figure S10: SEM images of different PEG-SH/PEG-Mal/Pep hydrogels. Figure S11: SEM images of PEG-SH/PEG-Mal/FKG hydrogels at different FKG:PEG-Mal ratios. Figure S12: Removing of the FKG peptide inside the hydrogels by the dialysis. Figure S13: Cell culture and viability of HAMSC cells on the PEG-SH/PEG-Mal/Pep hydrogels after incubation for 24 h. Figure S14: Cell culture and viability of HuH-7 cells on the PEG-SH/PEG-Mal/Pep hydrogels after incubation for 60 h. Figure S15: Determination of the reaction kinetics of PEG-SH and PEG-Mal. Figure S16: Summary of reaction and gelation rates of the 4-armed PEG-Mal and 4-armed PEG-SH.
