# Supplementary material for: Regulating the Homogeneity of Thiol-Maleimide Michael-Type Addition-Based Hydrogels Using Amino Biomolecules
Source: Gels. 2021 Nov 11;7(4):206. doi: 10.3390/gels7040206 (PMC8628763; doi:10.3390/gels7040206)
Supplement: Supplementary file 1 [file gels-07-00206-s001.zip › gels-1458868-supplementary.pdf]

Supplementary Materials for

Regulating the homogeneity of thiol-maleimide Michael-type addition based hydrogels using amino Biomolecules

Yu Guo [1, †], Jie Gu [2, †], Yuxin Jiang [2], Yanyan Zhou [2], Zhenshu Zhu[2], Tingting Ma[2], Yuanqi Cheng[2], Zongzhou Ji[1], Yonghua Jiao[1, *], Bin Xue[2, *], Yi Cao[2, *]

1 College of Life and Health Sciences, Northeastern University, Shenyang 110819, PR China; e-mail@e-mail.com

2 Collaborative Innovation Center of Advanced Microstructures, National Laboratory of Solid State Microstructure, Key Laboratory of Intelligent Optical Sensing and Manipulation, Ministry of Education, Department of Physics, Nanjing University, Nanjing 210093, China; e-mail@e-mail.com

* Correspondence: jiaoyh@mail.neu.edu.cn; xuebinnju@nju.edu.cn; caoyi@nju.edu.cn

† These authors contributed equally to this work

Experimental section:

Swelling measurements: In a typical experiment, the hydrogel volume after the gelation was set as V_1. After swelling in ddH_2O for 24 h, the volume of the hydrogel was set as V_2. The swelling ratio was calculated by equation: (Ɛ) $=V_2/V_1$. All the experiments were performed at room temperature.

Water content measurements: The wet weight of the hydrogel was recorded as W_1 after swelling and the dry weight of the hydrogel after the lyophilization was recorded as W_2. The water content was calculated as $(W_1-W_2)/W_1\times100\%$.

Scanning electron microscope (SEM): SEM images were obtained using a Quanta Scanning Electron Microscope (Quata 200, FEI) at 20 kV. The samples were dialyzed in ddH_2O for 24 hours and lyophilized prior to the measurement.

Determination of the thiol amount using 5,5′-Dithiobis-(2-nitrobenzoic acid) (DTNB): The PEG-SH/PEG-Mal/Pep hydrogels were prepared and soaked in PBS solutions (10 mM, pH=7.4) containing DTNB (2 mg mL^{-1}) for 24 hours. The amount of free sulfone in the hydrogel was measured by monitoring the concentration of the reaction product of thiol and DTNB in the solutions, which exhibited the characteristic absorption peak at 412 nm.

Cell culture and viability: Huh7 cells were cultivated in DMEM medium (Gibco, USA). 10% fetal bovine serum and 1% penicillin & streptomycin were added into the medium. HAMSC cells were routinely grown in α-MEM culture medium supplemented with 10% FBS, and 1% antibiotics. All cells used in the cell viability test were before 5 passages number. For the cell viability determination, 60 ul gel was prepared in 96-well plate and allowed to swell in PBS (10 mM, pH=7.4) for 24 hours. Huh7 cells were seeded on each well at the density of 1×10^5 per well, and the incubation was performed at 37 ^oC and 5% CO_2 for 24 hours. HAMSC cells were seeded on each well at the same density and the incubation of cells were performed at 37 ^oC and 5% CO_2 for 60 hours. After the incubation, calcein AM and propidium iodide (PI) staining was used to evaluate the cell viability. Each well of 96-well plate was washed twice

using PBS. The calcein AM and propidium iodide (PI) (Thermo, USA) dye solution was mixed well and added into each well. The plate was incubation at 37 °C for 30 minutes before being imaged using a fluorescence microscope (IX73, OLYMPUS, USA).

Figures

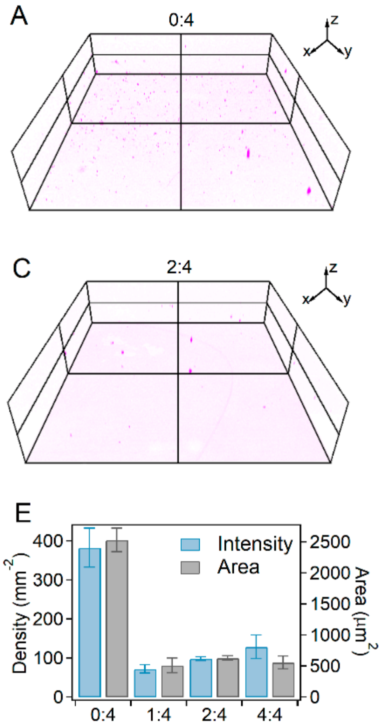

Figure S1 Spatial detection of unreacted thiol in PEG-Mal/PEG-SH/FKG hydrogels at different FKG:PEG-Mal ratios. (A-D) Spatial distribution of unreacted thiol in PEG-Mal/PEG-SH/FKG hydrogels using LCFM at FKG:PEG-Mal ratios of 0:4 (A), 1:4 (B), 2:4 (C) and 4:4 (D). The unreacted 4-armed PEG-SH was labeled with the thiol turn-on fluorescence probe. The red spots correspond to the locations of unreacted thiol, and the size of the scanning space was 1272 μm×1272 μm×300 μm. (F) Intensities and area of the fluorescent spots from the projected images of the three-dimensional constructs in the Z-axis direction for PEG-Mal/PEG-SH/FKG hydrogels at different FKG:PEG-Mal ratios. Values represent the mean and standard deviation (n=4-5).

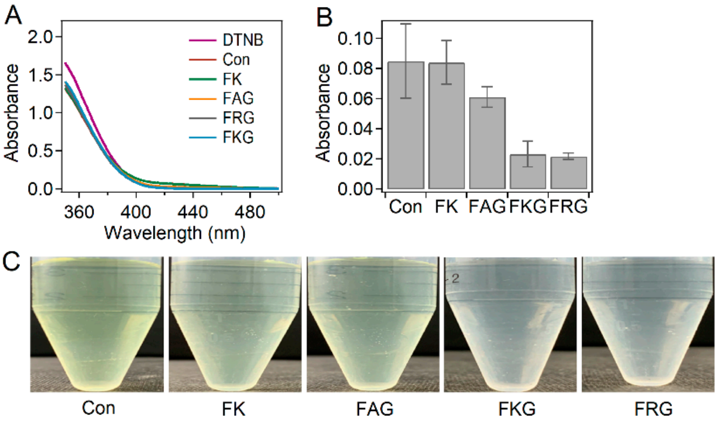

Figure S2 Detection of free thiol in PEG-Mal/PEG-SH/Pep hydrogels using DTNB. (A) UV absorbance of the DTNB solutions containing different PEG-Mal/PEG-SH/Pep hydrogels. (B) $OD_{412\ nm}$ correspond to the DTNB solutions containing different PEG-Mal/PEG-SH/Pep hydrogels. (C) Optical images of the DTNB solutions containing different PEG-Mal/PEG-SH/Pep hydrogels.

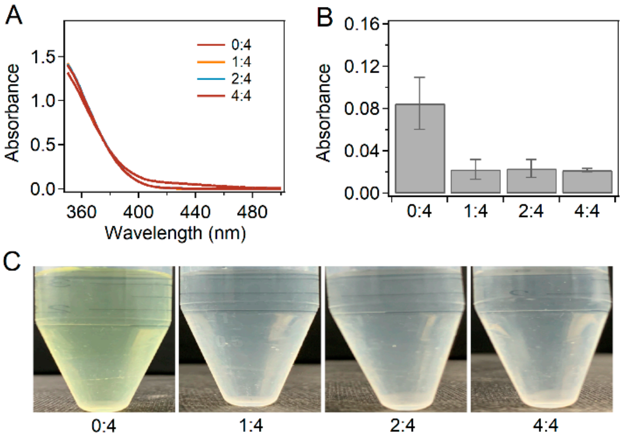

Figure S3 Detection of free thiol in PEG-Mal/PEG-SH/FKG hydrogels at different FKG:PEG-Mal ratios using DTNB. (A) UV absorbance of DTNB solutions containing PEG-Mal/PEG-SH/FKG hydrogels at different FKG:PEG-Mal ratios. (B) $OD_{412\ nm}$ correspond to leachates of PEG-Mal/PEG-SH/FKG hydrogels at different FKG:PEG-Mal ratios. (C) Optical images of the DTNB solutions containing PEG-Mal/PEG-SH/FKG hydrogels at different FKG:PEG-Mal ratios.

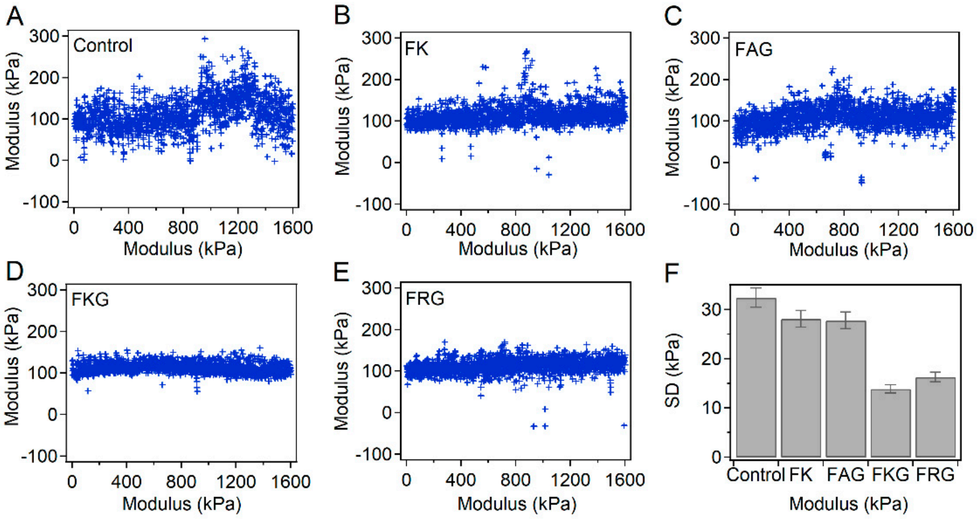

Figure S4 Typical scatter diagrams and standard deviation summary of Young's modulus from IT-AFM measurements for different PEG-Mal/PEG-SH/Pep hydrogels. (A-D) Typical scatter diagrams of Young's modulus quantified with IT-AFM for PEG-Mal/PEG-SH (A), PEG-Mal/PEG-SH/FK (B), PEG-Mal/PEG-SH/FAG (C), PEG-Mal/PEG-SH/FKG (D) and PEG-Mal/PEG-SH/FRG (E) hydrogels. (F) Standard deviation summary of Young's modulus from IT-AFM measurements for different PEG-Mal/PEG-SH/Pep hydrogels.

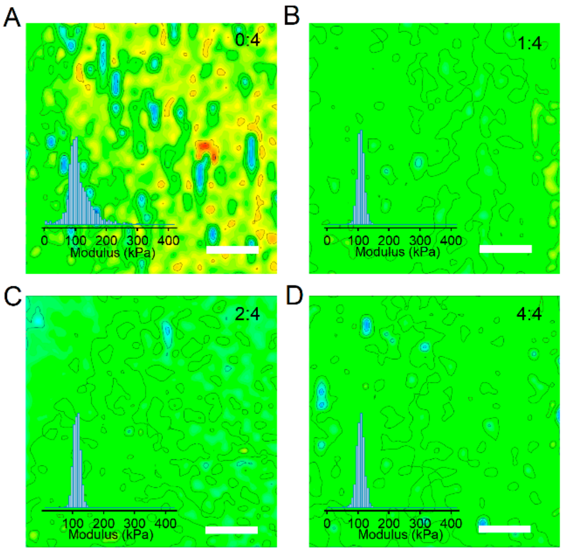

Figure S5 Mechanical homogeneity of the PEG-Mal/PEG-SH/FKG hydrogels at different FKG:PEG-Mal ratios. (A-B) Two-dimensional Young's modulus distributions of hydrogel surfaces determined by AFM for PEG-Mal/PEG-SH/FKG hydrogels at the FKG:PEG-Mal ratio of 0:4 (A), 1:4 (B), 2:4 (C) and 4:4 (D). The scale bar is 1.0 μm. Insets correspond to the histograms of Young's modulus.

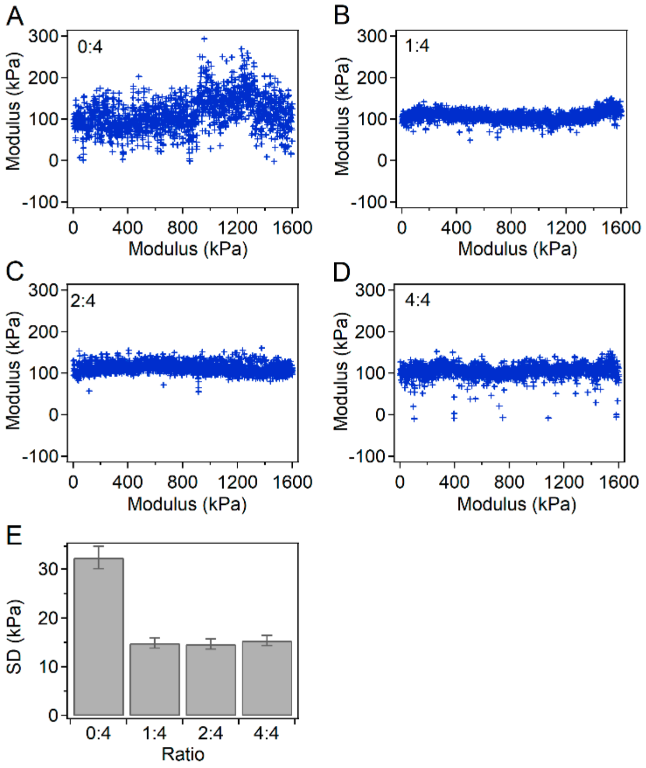

Figure S6 Typical scatter diagrams and standard deviation summary of Young's modulus from IT-AFM measurements for PEG-Mal/PEG-SH/FKG hydrogels at different FKG:PEG-Mal ratios. (A-D) Typical scatter diagrams of Young's modulus quantified with IT-AFM for PEG-Mal/PEG-SH/FKG hydrogels at the FKG:PEG-Mal ratios of 0:4 (A), 1:4 (B), 2:4 (C), and 4:4 (D). (E) Standard deviation summary of Young's modulus from IT-AFM measurements for PEG-Mal/PEG-SH/FKG hydrogels at different FKG:PEG-Mal ratios.

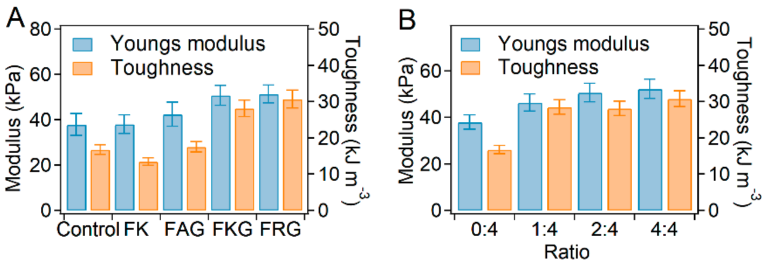

Figure S7 Summarized Young's modulus and toughness of the PEG-SH/PEG-Mal/Pep hydrogels in the compressive measurements. (A) Young's modulus and toughness of different PEG-SH/PEG-Mal/Pep hydrogels. (B) Young's modulus and toughness of PEG-SH/PEG-Mal/FKG hydrogels prepared at different FKG:PEG-Mal ratios.

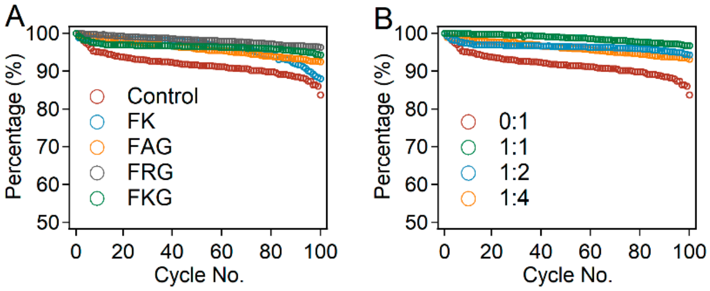

Figure S8 Normalized maximum stress recovery of hydrogels in 100 compression-relaxation cycles. (A) Normalized maximum stress for different PEG-SH/PEG-Mal/Pep hydrogels in 100 compression-relaxation cycles. (B) Normalized maximum stress for PEG-SH/PEG-Mal/FKG hydrogels at different FKG:PEG-Mal ratios in 100 compression-relaxation cycles.

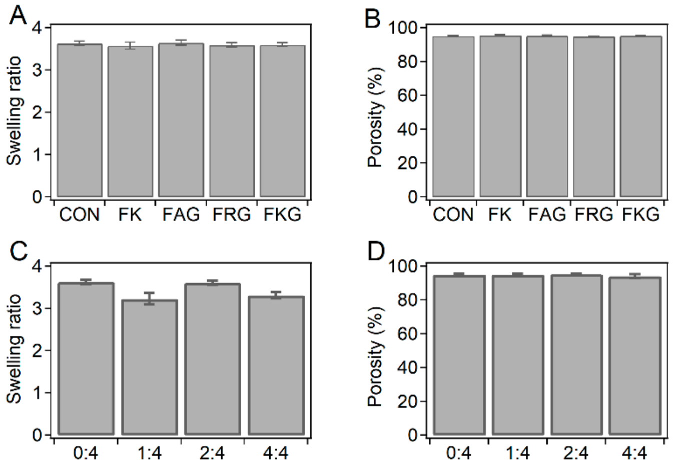

Figure S9 Swelling ratios and porosities of different hydrogels. (A-B) Swelling ratios (A) and porosities (B) of PEG-SH/PEG-Mal/Pep hydrogels. (C-D) Swelling ratios (C) and porosities (D) of PEG-SH/PEG-Mal/FKG hydrogels at different ratios of FKG and PEG-Mal.

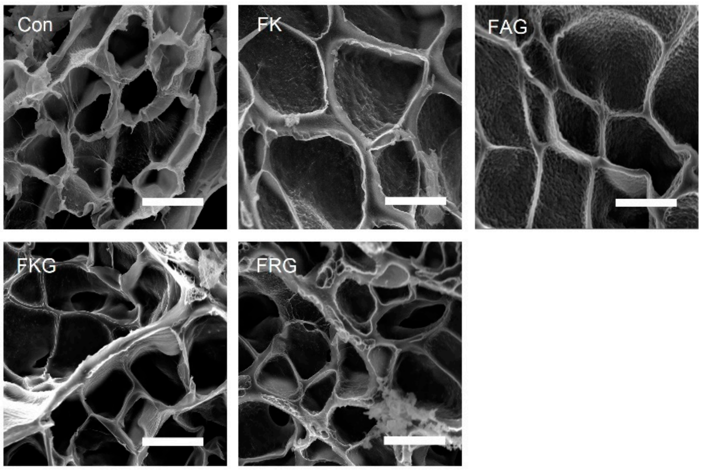

Figure S10 SEM images of different PEG-SH/PEG-Mal/Pep hydrogels (Control, FK, FAG, FKG and FRG). PEG-SH/PEG-Mal was set as control. Scale bar=50 μm.

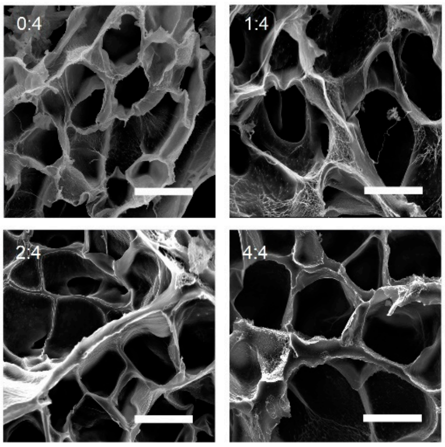

Figure S11 SEM images of PEG-SH/PEG-Mal/FKG hydrogels at different FKG:PEG-Mal ratios (0:4, 1:4, 2:4, and 4:4). Scale bar=50 μm.

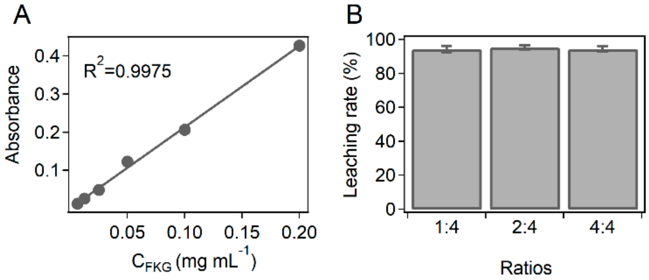

Figure S12 Removing of the FKG peptide inside the hydrogels by the dialysis. (A) Calibration curves of peptide concentration and OD_{280nm}. (B) Accumulated peptide removing percentages in the PEG-SH/PE-Mal/FKG hydrogels at different FKG:PEG-Mal ratios after three dialysis cycles.

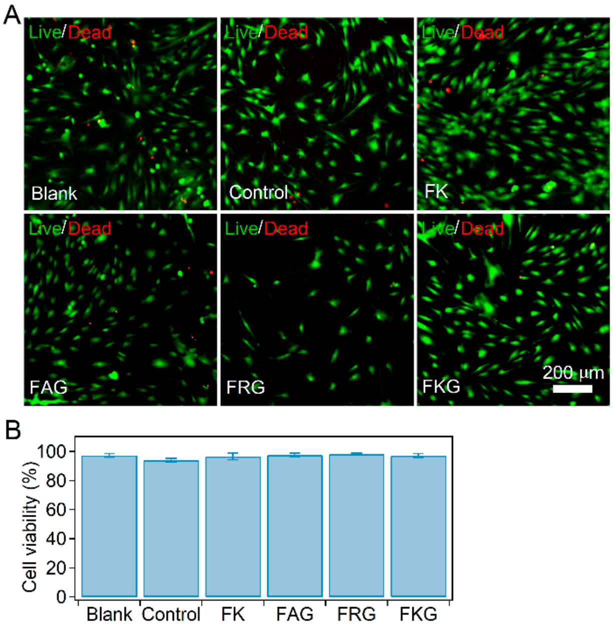

Figure S13 Cell culture and viability of HAMSC cells on the PEG-SH/PEG-Mal/Pep hydrogels after incubation for 60 hours. (A) Live/dead staining of HAMSC cells cultured on different PEG-SH/PEG-Mal/Pep hydrogels. Green color represented live cells with high enzymatic activity indicated by calcein AM. Red color of PI represented dead cells with compromised membranes. The cells cultured on the cell culture plates and PEG-SH/PEG-Mal hydrogels were set as the blank and control group, respectively. (B) Cell viability of HAMSC cells on the hydrogels determined by the live/dead staining.

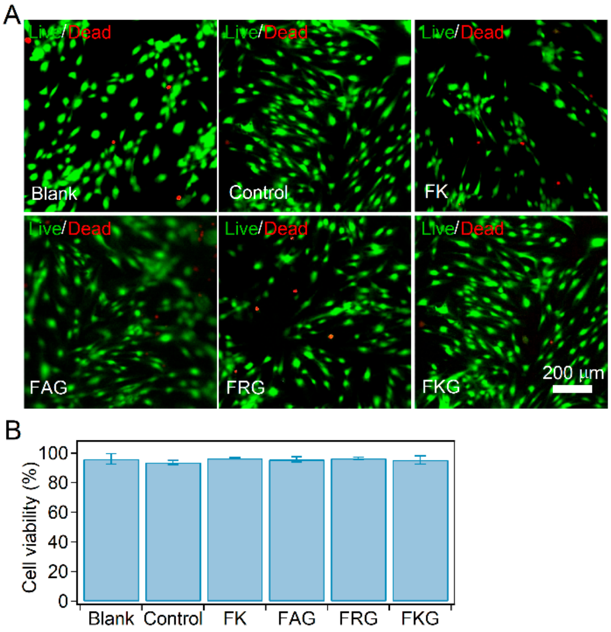

Figure S14 Cell culture and viability of Huh7 cells on the PEG-SH/PEG-Mal/Pep hydrogels after incubation for 24 hours. (A) Live/dead staining of Huh7 cells cultured on different PEG-SH/PEG-Mal/Pep hydrogels. Green color represented live cells with high enzymatic activity

indicated by calcein AM. Red color of PI represented dead cells with compromised membranes. The cells cultured on the cell culture plates and PEG-SH/PEG-Mal hydrogels were set as the blank and control group, respectively. (B) Cell viability of Huh7 cells on hydrogels determined by the live/dead staining.

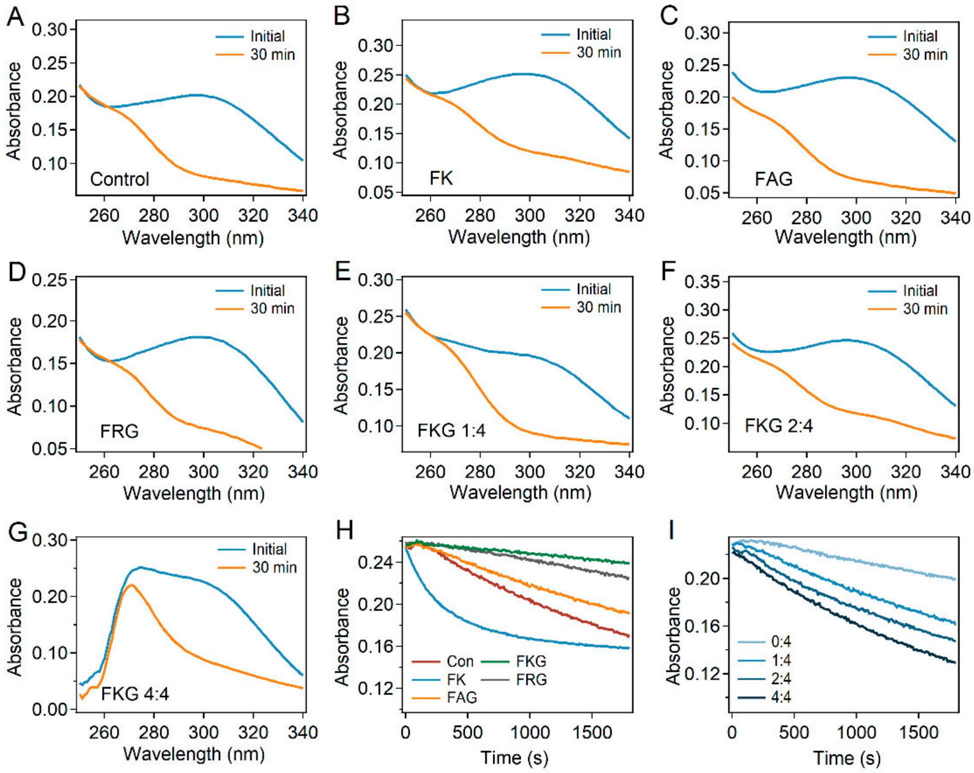

Figure S15 Determination of the reaction kinetics of PEG-SH and PEG-Mal. (A-D) UV spectra of PEG-Mal (0.4 mM) and PEG-SH (0.4 mM) mixtures before and after reacting for 30 minutes in the presence of different peptides (FK, FAG and FRG, peptide:PEG-Mal ratios = 2:4). (E-G) UV spectra of PEG-Mal (0.4 mM) and PEG-SH (0.4 mM) mixtures before and after reacting for 30 minutes at different FKG:PEG-Mal ratios (1:4, 2:4 and 4:4). (H) Time-dependent UV absorbance at 300 nm for mixtures of PEG-Mal (0.4 mM) and PEG-SH (0.4 mM) in the presence of different peptides (peptide:PEG-Mal ratio = 2:4). (I) Time-dependent UV absorbance at 300 nm for mixtures of PEG-Mal (0.4 mM) and PEG-SH (0.4 mM) at different ratios of FKG:PEG-Mal (0:1, 1:1, 2:1, and 4:1).

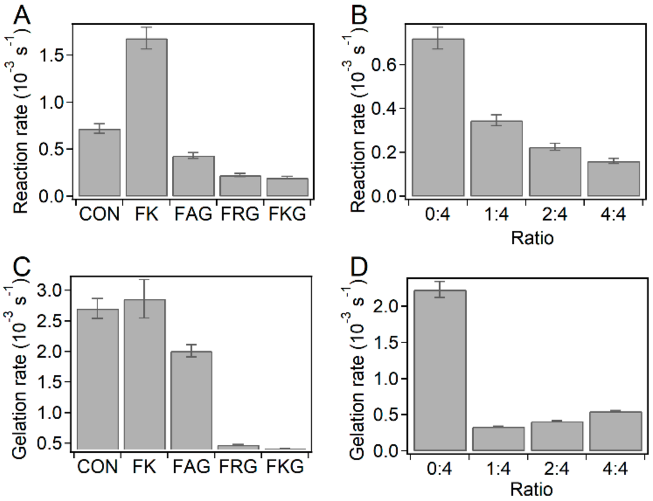

Figure S16 Summary of reaction and gelation rates of PEG-Mal and PEG-SH. (A-B) Reaction rates of PEG-Mal and PEG-SH during the first 400 seconds in the presence of different peptides (A), and at different FKG:PEG-Mal ratios (B). (C-D) Gelation rates of PEG-Mal and PEG-SH in the presence of different peptides (C), and at different FKG:PEG-Mal ratios (D).

Reference:

[1]. Hong, V.; Kislukhin, A. A.; Finn, M. G., Thiol-Selective Fluorogenic Probes for Labeling and Release. *J. Am. Chem. Soc.* **2009**, 131, 9986-9994.
